# The associations of eating behavior and dietary intake with metabolic syndrome in Japanese: Saku cohort baseline study

**DOI:** 10.1186/s40101-020-00250-w

**Published:** 2020-12-14

**Authors:** Akemi Morita, Naomi Aiba, Motohiko Miyachi, Shaw Watanabe, for the Saku Cohort Study Group

**Affiliations:** 1 Department of Public Health and Occupational Medicine, Mie University Graduate School of Medicine, 2-174 Edobashi, Tsu, Mie 5148507 Japan; 2 Department of Nutrition and Life Science, Kanagawa Institute of Technology, Kanagawa, 243-0292 Japan; 3 Department of Physical Activity Research, National Institutes of Biomedical Innovation, Health and Nutrition, Tokyo, 162-8636 Japan; 4 Life Science Promoting Association, Tokyo, 160-0015 Japan

**Keywords:** Metabolic syndrome (MetS), Dietary intake, Eating behavior, Baseline study

## Abstract

**Background:**

The prevention of metabolic syndrome (MetS) is a major public health concern in Japan. The effects of the relationship between eating behavior and nutritional intake on MetS remained unclear.

To evaluate nutrition’s role in preventing or exacerbating MetS, we examined the associations among eating behavior, nutritional intake, and MetS for the baseline study in the cohort subjects undergone health checkups.

**Methods:**

Four thousand and four hundred forty-seven Japanese men and women were enrolled at the Saku Central Hospital. They received an anthropometric and clinical examination and were assessed for present illness, lifestyle factors such as physical activity, smoking, drinking, and dietary habits at the enrollment. Eating behavior was analyzed by the Sakata’s Eating Behavior Questionnaire. Dietary assessment was made using a brief self-administered diet history questionnaire. Two thousand and six hundred two men and 1844 women aged more than 20 were analyzed.

**Results:**

The mean age in men and women were 59.2 and 58.4 years old and the mean body mass index (BMI) were 23.7 and 22.3 kg/m^2^, respectively. The percentages of MetS were 20.6 in men and 6.1 in women. In some nutrients, significantly higher energy-adjusted intakes in subjects without MetS than with Mets appeared both in men and women after age adjustment. After adjusting by age, energy-adjusted intake beverages in men and cereals in women were significantly higher in subjects with MetS than those without MetS. The scores of all the categories in eating behavior were significantly worse in subjects with MetS than those without MetS.

**Conclusions:**

The differences in dietary intake between subjects with Mets and without Mets were relatively small. The scores of all the categories in eating behavior were worse in subjects with MetS than without MetS. It was suggested that the problem lay in the quality of diet, not in the quantity, caused by bad eating habits. The potential influence of eating behavior and nutritional intake on MetS was presented in men and women.

## Background

Metabolic syndrome (MetS) is a cluster of cardiovascular risk factors, including abdominal obesity, hypertension, dyslipidemia, and glucose intolerance [1, 2]. The importance of MetS lies in its associated risk of cardiovascular disease and type 2 diabetes. The prevention of MetS and type 2 diabetes is a major challenge in Health Japan 21 [3]. The available scientific evidence on the associations between lifestyle modifications and MetS and its components is reviewed to derive recommendations for MetS prevention and management [4]. Especially, dietary habits change are the main therapeutic strategy for the treatment and management of obesity and MetS, but the most effective dietary pattern for its management has not been unclear [5].

The diet consumed by the Japanese, so-called Washoku, is widely perceived to be healthy owing to the low prevalence of coronary artery disease and relatively good life expectancy of the Japanese [6, 7]. However, studies have consistently determined that compliance with the Japanese healthy eating guidelines (Japanese Food Guide Spinning Top) is simultaneously associated with both favorable aspects of dietary intake such as higher intake of dietary fiber and micronutrients, and unfavorable aspects such as higher intake of saturated fats and sodium [8, 9]. Moreover, these large prospective cohort studies showed an inverse association between compliance with these guidelines and the major causes of mortality. A recent systematic review of Japanese studies that obtained dietary patterns presented that the food groups which contribute to dietary patterns termed healthy (vegetables, including potatoes, mushrooms, and seaweeds; fruits; soy products) are reasonably consistent with those often observed in Western countries [10].

To prevent MetS and subsequent diseases such as type 2 diabetes, it is required to elucidate the Japanese healthy diet’s component and pattern and its effect on MetS.

A series of epidemiological and clinical studies was carried out in Saku, Nagano Prefecture in Japan. Since 1990, a population-based Japan Public Health Center cohort study, consisting of 40–59-year-old residents, has been conducted in Saku [11]. According to the prevention of MetS, we established a clinical study (Saku Control Obesity Program (SCOP)) and compared the efficacy of SCOP intervention (i.e., change in the rate of metabolic syndrome before and after the intervention) [12–15]. In Saku Health Dock Center each year, about 7000 examinees have come to the center for a health checkup, including an oral glucose tolerance test (OGTT) by 75 g glucose intake, endoscopy, and recently abdominal CT, in addition to the routine laboratory test and physical checkups. The Saku Health Dock Center database contains approximately 196,000 records connecting to the hospital database. Our intervention and cohort study represented an approach incorporating extensive biological markers as health screening data, blood, urine, and gene storage for future analyses. We selected this area because of a long collaborative history for the primary prevention of chronic diseases.

To evaluate nutrition’s role in preventing or exacerbating MetS, we examined the associations among eating behavior, nutritional intake, and MetS for the baseline study in the cohort subjects undergone health checkups in the Saku Health Dock Center.

## Methods

### Study design

We performed a cross-sectional study using the baseline data of the Saku Cohort Study for Healthy Aging launched in 2009 at Saku Central Hospital, which is one of the core hospitals in Nagano Prefecture, Japan. The Saku Cohort Study has been described elsewhere [16]. In brief, this cohort study was designed to determine the risk factors for chronic diseases, including type 2 diabetes, cardiovascular disease, and cancer, among the community residents in Nagano Prefecture.

### Study population

Participants who visited Saku Central Hospital for a health checkup between May 2009 and March 2013, and who agreed to participate in the cohort, were included in the study. From the study population at baseline (*n* = 4447), we excluded one subject aged < 20 years old. The remaining 4446 participants (2602 men and 1844 women) were included for analysis.

### Measures

The general health screening included demographic characteristics (e.g., age and gender), medical histories, and physical and clinical examination. Besides, we assessed the participants’ physical activity levels, and all participants completed self-administered questionnaires regarding diet and mental health.

### Physical and clinical examination

Each participant underwent all the examinations on the same morning after an overnight fast (12 h). The height (cm) and weight (kg) of the subject were measured with an automatic scale (Tanita, BF-220, Tokyo, Japan), in light clothing. The body mass index (BMI) was calculated as the weight (kg) divided by the squared height (m^2^). Waist circumference was measured twice at the umbilicus level while the subject was in a standing position using a fiberglass measuring tape; the average measurement was used for the analysis. Blood pressure was measured while the subject was in a sitting position using a validated automated blood pressure monitor (ES-H55; Terumo, Tokyo, Japan). Blood pressure was measured twice to calculate the average of the two values for the analysis.

About half of the participants were assessed the cross-sectional area of visceral fat tissue (visceral fat area: VFA) based on a computed tomography (CT) scan at the level of the umbilicus while the subject was in a supine position (Fat scan; N2 system Corp., Japan).

Blood samples were collected for the measurement of fasting plasma glucose, insulin, hemoglobin A1c (HbA1c), serum total cholesterol, high-density lipoprotein (HDL) cholesterol, low-density lipoprotein (LDL) cholesterol; and triglyceride, γ-glutamyl transferase, uric acid, blood urea nitrogen, and creatinine levels.

The values for HbA1c were collected as Japan Diabetes Society (JDS) values, and then converted to National Glycohemoglobin Standardization Program (NGSP) values using the following conversion formula: HbA1c (NGSP) = 1.02 × HbA1c (JDS) + 0.25%. The homeostasis model assessment of insulin resistance (HOMA-IR) was calculated as follows: fasting insulin (μIU/ml) × fasting glucose (mg/dL)/405.

### Dietary intake and dietary habits

The previous month's dietary intake was assessed using a validated brief self-administered diet history questionnaire (BDHQ) [17]. The BDHQ is a structured questionnaire that inquiries about 58 food and beverage items; the specified serving sizes are described in terms of the natural portions or the standard weight and volume measurements of the servings commonly consumed in the general Japanese population. The intake of energy and nutrients, and food was calculated using an ad hoc computer program for BDHQ. The BDHQ is a short form of the comprehensive version of a validated self-administered diet history questionnaire (DHQ). The validation of the DHQ and BDHQ was performed using weighed dietary records or biological markers. The validity of the energy-adjusted intakes of many nutrients and food groups has been previously studied in the adult Japanese population [18].

Participants’ eating behavior was analyzed by a questionnaire established by Sakata in the Manual of Obesity 2006 written by the Japan Society for the Study of Obesity [19]. The questionnaire’s 55 statements are based on the statements given by obese people in a clinical survey, and subjects were asked to agree or disagree on a 4-point Likert scale: (1) disagree, (2) sometimes, (3) having a trend, and (4) agree. The questionnaire is assessed by categorizing 54 items into the following eight categories: (1) perception gap about constitution and weight, (2) motivation for eating, (3) substitute eating, (4) perception gap about feeling of fullness and hunger, (5) bad eating habits, (6) contents of diet, (7) eating pattern, and (8) total of all of them. Each category score was calculated by sex. One is a dummy question. The higher score in this questionnaire indicated worse in eating behavior (see Appendix 1).

### Metabolic syndrome

Metabolic syndrome was diagnosed consistent with the criteria of the Examination Committee for the Criteria for the Diagnosis of Metabolic Syndrome in Japan 2005 [20] The definition of metabolic syndrome was abdominal obesity with a waist circumference ≥ 85 cm for men and ≥ 90 cm for women and two or more of the following three risk factors: (1) high blood pressure (systolic blood pressure ≥ 130 mmHg and/or diastolic blood pressure ≥ 85 mmHg, or treatment for previously diagnosed hypertension), (2) hyperglycemia (fasting glucose ≥ 110 mg/dL or treatment for previously diagnosed type 2 diabetes), and (3) dyslipidemia (triglyceride levels ≥ 150 mg/dL and/or HDL-cholesterol < 40 mg/dL, or treatment for previously diagnosed dyslipidemia).

### Statistical analysis

Baseline characteristics were compared between sexes and between subjects with MetS and without MetS using the unpaired *t* test for continuous variables. Crude value and energy-adjusted value, which were calculated as the amount of food consumed divided by 1000 kcal (density model), of dietary intake were analyzed. Comparisons of dietary intake adjusted for confounding factors were performed by analysis of covariance (ANCOVA) when necessary. Pearson or Spearman correlation coefficients (*r*) were calculated to evaluate associations among physical and clinical findings, dietary intakes, and eating behavior. *P* < 0.05 was considered significant. Statistical analyses were performed using SPSS statistical software (Version 25.0 J; IBM Japan Inc., Tokyo).

## Results

In total, 4447 Japanese men and women participated in our cohort. In this study, 2602 men and 1844 women aged 20 and over were analyzed.

Table 1 shows the basic characteristics of the subjects. The mean age in men and women were 59.2 and 58.4 years old and the mean BMI were 23.7 and 22.3 kg/m^2^, respectively. The average waist circumference in men was 85.3 cm which met MetS criteria in Japan. Visceral fat areas were measured by CT in 1293 men and 841 women. Visceral obesity was 58.0% in men and 20.3% in women diagnosed according to the high visceral fat area (≥ 100 cm^2^).

**Table 1:** Basic characteristics of the subjects

	Men	Women
	(*n* = 2602)	(*n* = 1844)
Age (years)	59.2 ± 10.0	58.4 ± 9.8
Height (cm)	168.1 ± 6.1	155.6 ± 5.6
Weight (kg)	67.1 ± 9.7	54.1 ± 8.2
BMI (kg/m^2^)	23.7 ± 2.9	22.3 ± 3.2
Waist circumference (cm)	85.3 ± 8.0	80.7 ± 9.0
Visceral fat area (cm^2^)	114.1 ± 44.7	73.0 ± 36.7
Systolic blood pressure (mmHg)	119.9 ± 15.0	112.4 ± 15.3
Diastolic blood pressure (mmHg)	75.6 ± 11.1	69.4 ± 11.2
HDL cholesterol (mg/dL)	54.8 ± 13.5	64.4 ± 14.3
LDL cholesterol (mg/dL)	118.6 ± 28.8	122.7 ± 29.0
Triglycerides (mg/dL)	125.0 ± 77.3	92.5 ± 49.7
γ-glutamyl transferase (IU/L)	47.9 ± 51.8	23.3 ± 22.0
Uric acid (mg/dL)	6.1 ± 1.2	4.7 ± 1.0
Blood urea nitrogen (mg/dL)	14.7 ± 3.6	13.8 ± 3.3
Creatinine (mg/dL)	0.86 ± 0.18	0.64 ± 0.11
Fasting plasma glucose (mg/dL)	104.7 ± 17.5	99.0 ± 14.6
HbA1c (%)	5.7 ± 0.6	5.7 ± 0.5
Fasting insulin (μU/mL)	5.1 ± 3.6	4.6 ± 2.8
HOMA-IR	1.4 ± 1.2	1.2 ± 0.9

Values are means ± SD

Table 2 shows daily nutritional intake in men and women. Macronutrients and major micronutrients which were possible to affect metabolic risk were presented in Table 2 [5–10]. Energy and crude nutrient intakes excepting potassium, calcium, and vitamin D were significantly higher in men than in women. However, all energy-adjusted nutrients intakes were significantly higher in women than in men.

**Table 2:** Daily nutritional intake of the subjects

		Crude			Energy-adjusted	
		Men (*n* = 2602)	Women (*n* = 1844)		Men (*n* = 2602)	Women (*n* = 1844)
Energy	(kcal)	2380 ± 683	1933 ± 515*			
Protein	(g)	89.7 ± 33.6	80.5 ± 28.0*	(g/1000 kcal)	37.5 ± 7.3	41.4 ± 7.5*
Animal protein	(g)	52.4 ± 26.1	48.0 ± 22.1*	(g/1000 kcal)	21.7 ± 7.4	24.5 ± 7.9*
Plant protein	(g)	37.2 ± 11.4	32.5 ± 9.2*	(g/1000 kcal)	15.7 ± 2.6	16.9 ± 2.4*
Fat	(g)	63.8 ± 24.2	58.9 ± 20.7*	(g/1000 kcal)	26.7 ± 6.0	30.2 ± 5.6*
Animal fat	(g)	29.6 ± 13.8	27.2 ± 11.4*	(g/1000 kcal)	12.3 ± 4.0	13.9 ± 3.9*
Plant fat	(g)	34.1 ± 13.7	31.8 ± 12.6*	(g/1000 kcal)	14.3 ± 4.1	16.3 ± 4.4*
Saturated fatty acid	(g)	15.9 ± 6.5	15.1 ± 5.6*	(g/1000 kcal)	6.6 ± 1.8	7.7 ± 1.7*
n-3 polyunsaturated fatty acid	(g)	3.7 ± 1.7	3.4 ± 1.5*	(g/1000 kcal)	1.5 ± 0.5	1.7 ± 0.5*
n-6 polyunsaturated fatty acid	(g)	13.0 ± 4.9	11.6 ± 4.4*	(g/1000 kcal)	5.5 ± 1.4	6.0 ± 1.5*
Carbohydrate	(g)	313 ± 96	259 ± 70*	(g/1000 kcal)	132 ± 20	135 ± 17*
Soluble dietary fiber	(g)	4.0 ± 1.8	4.1 ± 1.6*	(g/1000 kcal)	1.7 ± 0.6	2.1 ± 0.6*
Insoluble dietary fiber	(g)	11.5 ± 4.5	11.5 ± 4.3*	(g/1000 kcal)	4.9 ± 1.4	6.0 ± 1.6*
Total dietary fiber	(g)	16.1 ± 6.5	16.1 ± 6.1*	(g/1000 kcal)	6.8 ± 2.1	8.3 ± 2.3*
Sodium	(mg)	5516 ± 1689	4790 ± 1438*	(mg/1000 kcal)	2358 ± 493	2505 ± 500*
Salt equivalent	(g)	13.9 ± 4.3	12.1 ± 3.6*	(g/1000 kcal)	5.9 ± 1.2	6.3 ± 1.3*
Potassium	(mg)	3380 ± 1287	3317 ± 1184	(mg/1000 kcal)	1423 ± 372	1713 ± 410*
Calcium	(mg)	701 ± 298	687 ± 275	(mg/1000 kcal)	296 ± 95	354 ± 102*
Magnesium	(mg)	337 ± 117	310 ± 103*	(mg/1000 kcal)	142 ± 29	160 ± 32*
Vitamin D	(μg)	21.6 ± 15.1	21.0 ± 14.0	(μg/1000 kcal)	8.9 ± 5.0	10.6 ± 5.8*
Vitamin B1	(mg)	0.97 ± 0.36	0.93 ± 0.31*	(mg/1000 kcal)	0.40 ± 0.09	0.48 ± 0.09*
Vitamin B2	(mg)	1.68 ± 0.61	1.55 ± 0.51*	(mg/1000 kcal)	0.71 ± 0.18	0.80 ± 0.17*

Values are crude and energy-adjusted means ± SD

*Significant difference between sexes (*p* < 0.05)

The following analyses were conducted by sex.

**Fig. 1:**
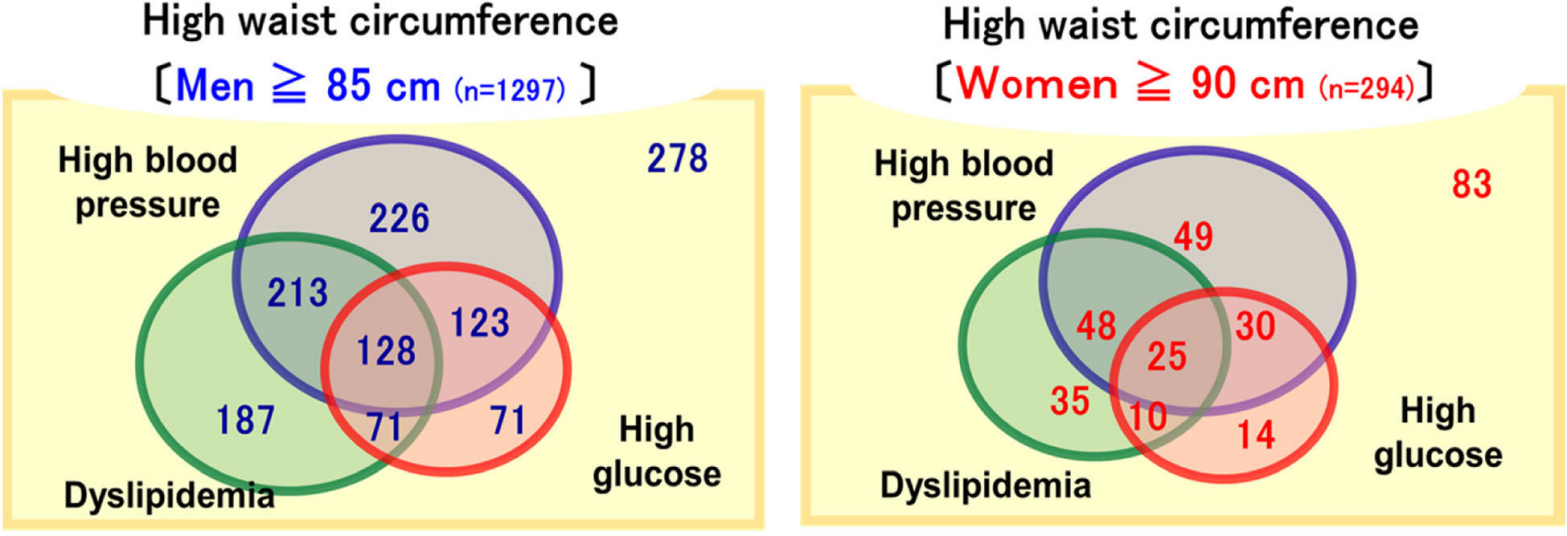
Prevalence of diseases according to MetS criteria. Values in the figure are numbers of subjects who met the criteria of each disease according to MetS. The number of subjects with MetS was 535 (20.6%) in men and 113 (6.1%) in women. MetS: metabolic syndrome

The percentage of MetS was higher in men (20.6%) than in women (6.1%). Figure 1 shows the prevalence of diseases according to “Metabolic Syndrome Criteria”, as high blood pressure, high glucose, and dyslipidemia. The numbers of high blood pressure, high glucose, and dyslipidemia were 842 (men 690, women 152), 472 (men 393, women 79), and 717 (men 599, women 118), respectively. The subjects with MetS were older and had worse anthropometric and clinical characteristics than those without MetS in both men and women (Table 3).

**Table 3:** Associations between basic characteristics and MetS

	Men		Women	
	MetS (*n* = 535)	w/o MetS (*n* = 2067)	MetS (*n* = 113)	w/o MetS (*n* = 1731)
Age (years)	61.1 ± 8.9	58.6 ± 10.2*	63.1 ± 8.4	58.1 ± 9.8*
Height (cm)	168.4 ± 5.8	168.0 ± 6.2	154.4 ± 5.4	155.7 ± 5.6*
Weight (kg)	74.2 ± 9.1	65.2 ± 9.0*	66.0 ± 7.7	53.3 ± 7.7*
BMI (kg/m^2^)	26.1 ± 2.7	23.1 ± 2.6*	27.7 ± 2.9	22.0 ± 2.9*
Waist circumference (cm)	92.6 ± 6.3	83.4 ± 7.3*	**96.7 ± 5.7**	79.7 ± 8.3*
Visceral fat area (cm^2^)	142.6 ± 46.0	104.4 ± 39.8*	115.6 ± 37.7	67.8 ± 33.1*
Systolic blood pressure (mmHg)	127.4 ± 14.9	117.6 ± 14.4*	127.8 ± 16.4	111.4 ± 14.7*
Diastolic blood pressure (mmHg)	80.4 ± 10.9	74.0 ± 10.8*	78.4 ± 11.6	68.9 ± 10.9*
HDL cholesterol (mg/dL)	49.2 ± 11.6	56.2 ± 13.6*	56.6 ± 12.1	64.9 ± 14.3*
LDL cholesterol (mg/dL)	118.0 ± 28.6	118.8 ± 28.9	130.2 ± 31.4	122.2 ± 28.8*
Triglycerides (mg/dL)	169.3 ± 97.7	113.5 ± 66.4*	137.3 ± 64.2	89.6 ± 47.2*
γ-glutamyl transferase (IU/L)	64.2 ± 67.1	43.7 ± 46.1*	37.1 ± 32.9	22.4 ± 20.8*
Uric acid (mg/dL)	6.4 ± 1.3	6.1 ± 1.2*	5.4 ± 1.2	4.6 ± 1.0*
Blood urea nitrogen (mg/dL)	14.8 ± 3.8	14.6 ± 3.6	14.1 ± 3.6	13.8 ± 3.3
Creatinine (mg/dL)	0.87 ± 0.17	0.85 ± 0.19*	0.64 ± 0.14	0.63 ± 0.11
Fasting plasma glucose (mg/dL)	116.2 ± 21.8	101.8 ± 14.8*	116.1 ± 24.6	97.9 ± 12.9*
HbA1c (%)	6.1 ± 0.7	5.7 ± 0.5*	6.2 ± 0.7	5.7 ± 0.5*
Fasting insulin (μU/mL)	7.6 ± 4.9	4.4 ± 2.9*	8.7 ± 4.5	4.4 ± 2.5*
HOMA-IR	2.2 ± 1.8	1.1 ± 0.8*	2.6 ± 1.7	1.1 ± 0.7*

Values are means ± SD. *MetS* metabolic syndrome, *w/o* without

*Significant difference between in the subject with MetS and without MetS (*p* < 0.05)

The association between dietary intake and MetS was presented in Tables 4 and 5. We compared nutritional intake between subjects with MetS and without MetS. Aside from sodium and vitamin D intake, both crude and energy-adjusted nutrients intakes were higher in subjects without MetS than with Mets in men. There were significant differences in some crude and energy-adjusted nutrient intake between men with MetS and without MetS. In women, there was no significant difference in all crude nutrients intakes between subjects with MetS and without MetS. However, women with MetS had significantly higher energy-adjusted sodium intake than those without MetS. After adjusting by age as a confounding factor, the association between sodium intake and MetS was not found both in men and women. In some nutrients, significantly higher energy-adjusted intakes in subjects without MetS than with Mets appeared both in men and women after age adjustment (data not shown).

**Table 4:** Associations between nutritional intake and MetS

**Men**		Crude			Energy adjusted	
		MetS	w/o MetS		MetS	w/o MetS
		(*n* = 535)	(*n* = 2067)		(*n* = 535)	(*n* = 2067)
Energy	(kcal)	2350 ± 694	2387 ± 680			
Protein	(g)	87.3 ± 32.1	90.3 ± 34.0	(g/1000 kcal)	37.7 ± 7.4	37.6 ± 7.3†
Animal protein	(g)	51.3 ± 24.6	52.7 ± 26.5	(g/1000 kcal)	21.6 ± 7.4	21.8 ± 7.5
Plant protein	(g)	36.0 ± 11.2	37.5 ± 11.5*	(g/1000 kcal)	15.5 ± 2.7	15.8 ± 2.5*†
Fat	(g)	61.4 ± 23.5	64.4 ± 24.4*	(g/1000 kcal)	26.0 ± 6.3	26.8 ± 5.9*†
Animal fat	(g)	28.4 ± 12.9	30.0 ± 14.0*	(g/1000 kcal)	11.9 ± 3.9	12.4 ± 4.0*†
Plant fat	(g)	33.0 ± 13.6	34.4 ± 13.7*	(g/1000 kcal)	14.1 ± 4.3	14.4 ± 4.0
Saturated fatty acid	(g)	15.1 ± 6.2	16.1 ± 6.5*	(g/1000 kcal)	6.4 ± 1.7	6.7 ± 1.8*†
n-3 polyunsaturated fatty acid	(g)	3.7 ± 1.7	3.7 ± 1.7	(g/1000 kcal)	1.6 ± 0.5	1.5 ± 0.5
n-6 polyunsaturated fatty acid	(g)	12.5 ± 4.8	13.1 ± 5.0*	(g/1000 kcal)	5.4 ± 1.5	5.5 ± 1.4*†
Carbohydrate	(g)	302 ± 98	316 ± 96*	(g/1000 kcal)	129 ± 22	133 ± 19*†
Soluble dietary fiber	(g)	3.8 ± 1.7	4.0 ± 1.8*	(g/1000 kcal)	1.6 ± 0.6	1.7 ± 0.6†
Insoluble dietary fiber	(g)	11.1 ± 4.4	11.6 ± 4.5*	(g/1000 kcal)	4.8 ± 1.5	4.9 ± 1.4†
Total dietary fiber	(g)	15.5 ± 6.3	16.2 ± 6.6*	(g/1000 kcal)	6.7 ± 2.2	6.8 ± 2.1†
Sodium	(mg)	5525 ± 1701	5514 ± 1687	(mg/1000 kcal)	2395 ± 505	2349 ± 490
Salt equivalent	(g)	13.9 ± 4.3	13.9 ± 4.3	(g/1000 kcal)	6.0 ± 1.3	5.9 ± 1.2
Potassium	(mg)	3274 ± 1244	3407 ± 1297*	(mg/1000 kcal)	1398 ± 376	1430 ± 371†
Calcium	(mg)	683 ± 292	706 ± 299	(mg/1000 kcal)	291 ± 94	297 ± 95†
Magnesium	(mg)	330 ± 114	339 ± 118	(mg/1000 kcal)	141 ± 29	142 ± 29†
Vitamin D	(μg)	21.9 ± 15.3	21.6 ± 15.1	(μg/1000 kcal)	9.1 ± 5.0	8.8 ± 5.0
Vitamin B1	(mg)	0.93 ± 0.33	0.97 ± 0.37*	(mg/1000 kcal)	0.39 ± 0.09	0.41 ± 0.09*†
Vitamin B2	(mg)	1.64 ± 0.57	1.69 ± 0.62	(mg/1000 kcal)	0.70 ± 0.17	0.71 ± 0.18†
**Women**		Crude			Energy adjusted	
		MetS (*n* = 113)	w/o MetS (*n* = 1731)		MetS (*n* = 113)	w/o MetS (*n* = 1731)
Energy	(kcal)	1880 ± 519	1937 ± 514			
Protein	(g)	78.8 ± 27.5	80.6 ± 28.0	(g/1000 kcal)	41.9 ± 8.1	41.3 ± 7.5
Animal protein	(g)	47.3 ± 21.8	48.0 ± 22.0	(g/1000 kcal)	25.0 ± 8.3	24.4 ± 7.8
Plant protein	(g)	31.5 ± 8.8	32.6 ± 9.2	(g/1000 kcal)	16.9 ± 2.4	16.9 ± 2.4
Fat	(g)	56.1 ± 21.1	59.1 ± 20.7	(g/1000 kcal)	29.4 ± 5.8	30.2 ± 5.5
Animal fat	(g)	26.4 ± 11.5	27.2 ± 11.4	(g/1000 kcal)	13.9 ± 4.0	13.9 ± 3.9
Plant fat	(g)	29.7 ± 12.2	31.9 ± 12.6	(g/1000 kcal)	15.6 ± 4.3	16.3 ± 4.4
Saturated fatty acid	(g)	14.4 ± 5.7	15.1 ± 5.6	(g/1000 kcal)	7.6 ± 1.7	7.7 ± 1.7
n-3 polyunsaturated fatty acid	(g)	3.3 ± 1.5	3.4 ± 1.5	(g/1000 kcal)	1.7 ± 0.5	1.7 ± 0.5
n-6 polyunsaturated fatty acid	(g)	11.1 ± 4.3	11.6 ± 4.4	(g/1000 kcal)	5.8 ± 1.5	6.0 ± 1.5
Carbohydrate	(g)	254 ± 69	260 ± 70	(g/1000 kcal)	136 ± 19	135 ± 17
Soluble dietary fiber	(g)	3.8 ± 1.4	4.1 ± 1.6	(g/1000 kcal)	2.1 ± 0.6	2.1 ± 0.6†
Insoluble dietary fiber	(g)	11.2 ± 4.0	11.5 ± 4.3	(g/1000 kcal)	6.0 ± 1.5	6.0 ± 1.6
Total dietary fiber	(g)	15.7 ± 5.7	16.2 ± 6.1	(g/1000 kcal)	8.4 ± 2.2	8.3 ± 2.3
Sodium	(mg)	4817 ± 1355	4788 ± 1444	(mg/1000 kcal)	2618 ± 534	2497 ± 497*
Salt equivalent	(g)	12.2 ± 3.4	12.1 ± 3.6	(g/1000 kcal)	6.6 ± 1.4	6.3 ± 1.3*
Potassium	(mg)	3161 ± 1105	3327 ± 1189	(mg/1000 kcal)	1694 ± 406	1714 ± 410†
Calcium	(mg)	664 ± 261	688 ± 276	(mg/1000 kcal)	355 ± 105	354 ± 102
Magnesium	(mg)	300 ± 99	311 ± 104	(mg/1000 kcal)	161 ± 33	160 ± 32†
Vitamin D	(μg)	20.8 ± 13.6	21.0 ± 14.0	(μg/1000 kcal)	10.9 ± 5.7	10.6 ± 5.8
Vitamin B1	(mg)	0.90 ± 0.31	0.93 ± 0.31	(mg/1000 kcal)	0.48 ± 0.09	0.48 ± 0.09
Vitamin B2	(mg)	1.52 ± 0.50	1.56 ± 0.51	(mg/1000 kcal)	0.82 ± 0.19	0.80 ± 0.17

Values are crude and energy-adjusted means ± SD. *MetS* metabolic syndrome, *w/o* without

*Significant difference in crude or energy-adjusted nutrient intake (/1000 kcal) between in the subject with MetS and without MetS (*p* < 0.05)

†Significant difference in energy-adjusted nutrient intake (/1000 kcal) between in the subject with MetS and without MetS adjusted by age (*p* < 0.05)

**Table 5:** Associations between Food Group Intake and MetS

**Men**		Crude			Energy adjusted	
		MetS (*n* = 535)	w/o MetS (*n* = 2067)		MetS (*n* = 535)	w/o MetS (*n* = 2067)
Cereals	(g)	533 ± 200	553 ± 202*	(g/1000 kcal)	231 ± 71	234 ± 63
Potatoes	(g)	76 ± 69	82 ± 64	(g/1000 kcal)	31 ± 25	33 ± 24†
Bean and soybean product	(g)	77 ± 46	83 ± 50*	(g/1000 kcal)	33 ± 19	35 ± 19†
Green and yellow vegetables	(g)	126 ± 81	136 ± 87*	(g/1000 kcal)	54 ± 35	57 ± 35†
Light-colored vegetables	(g)	217 ± 119	225 ± 130	(g/1000 kcal)	93 ± 48	94 ± 48
Fruits	(g)	115 ± 100	118 ± 100	(g/1000 kcal)	50 ± 43	50 ± 42
Fish and shellfish	(g)	126 ± 80	125 ± 83	(g/1000 kcal)	53 ± 26	51 ± 26
Meats	(g)	68 ± 40	76 ± 49 *	(g/1000 kcal)	29 ± 15	31 ± 16*†
Milk and dairy product	(g)	143 ± 107	154 ± 109*	(g/1000 kcal)	62 ± 45	66 ± 48†
Fat and oil	(g)	19 ± 12	19 ± 12	(g/1000 kcal)	8 ± 4	8 ± 5
Confectionery	(g)	39 ± 40	43 ± 41*	(g/1000 kcal)	16 ± 13	17 ± 14*†
Preference for beverages	(g)	1020 ± 488	950 ± 421*	(g/1000 kcal)	443 ± 192	414 ± 187*†
**Women**		Crude			Energy adjusted	
		MetS	w/o MetS		MetS	w/o MetS
		(*n* = 113)	(*n* = 1731)		(*n* = 113)	(*n* = 1731)
Cereals	(g)	403 ± 139	393 ± 129	(g/1000 kcal)	217 ± 61	207 ± 59†
Potatoes	(g)	69 ± 50	81 ± 59*	(g/1000 kcal)	37 ± 25	41 ± 27†
Bean and soybean product	(g)	73 ± 41	77 ± 45	(g/1000 kcal)	39 ± 20	40 ± 22
Green and yellow vegetables	(g)	140 ± 80	149 ± 88	(g/1000 kcal)	75 ± 41	77 ± 42
Light-colored vegetables	(g)	246 ± 123	252 ± 129	(g/1000 kcal)	130 ± 52	130 ± 59
Fruits	(g)	132 ± 89	137 ± 97	(g/1000 kcal)	72 ± 50	71 ± 48
Fish and shellfish	(g)	119 ± 71	116 ± 73	(g/1000 kcal)	63 ± 31	59 ± 30
Meats	(g)	59 ± 39	65 ± 38	(g/1000 kcal)	31 ± 17	33 ± 16
Milk and dairy product	(g)	147 ± 90	147 ± 93	(g/1000 kcal)	80 ± 50	77 ± 47
Fat and oil	(g)	16 ± 11	17 ± 12	(g/1000 kcal)	8 ± 5	9 ± 5
Confectionery	(g)	48 ± 35	54 ± 42	(g/1000 kcal)	25 ± 18	27 ± 18
Preference for beverages	(g)	678 ± 289	717 ± 319	(g/1000 kcal)	377 ± 174	384 ± 182

Values are crude and energy-adjusted means ± SD. *MetS* metabolic syndrome. *w/o* without

*Significant difference in crude or energy-adjusted food group intake (/1000 kcal) between in the subject with MetS and without MetS (*p* < 0.05)

†Significant difference in energy-adjusted food group intake (/1000 kcal) between in the subject with MetS and without MetS adjusted by age (*p* < 0.05)

In Table 5, the food group’s crude food intakes tended to be higher in subjects without MetS than with MetS, except beverages in men, cereals in women, and fishes in both sexes. In some crude food intakes, including beverages, there were significant differences between subjects with MetS and without MetS. Energy-adjusted food intake showed a similar trend in men. However, in women, energy-adjusted cereal and some other food intakes were slightly higher in subjects with MetS than without MetS. After adjusting by age, energy-adjusted intake beverages in men and cereals in women were significantly higher in subjects with MetS than without MetS. Energy-adjusted intake of some foods in men and potatoes in women showed significantly higher without MetS than with Mets after age adjustment (data not shown).

**Fig. 2:**
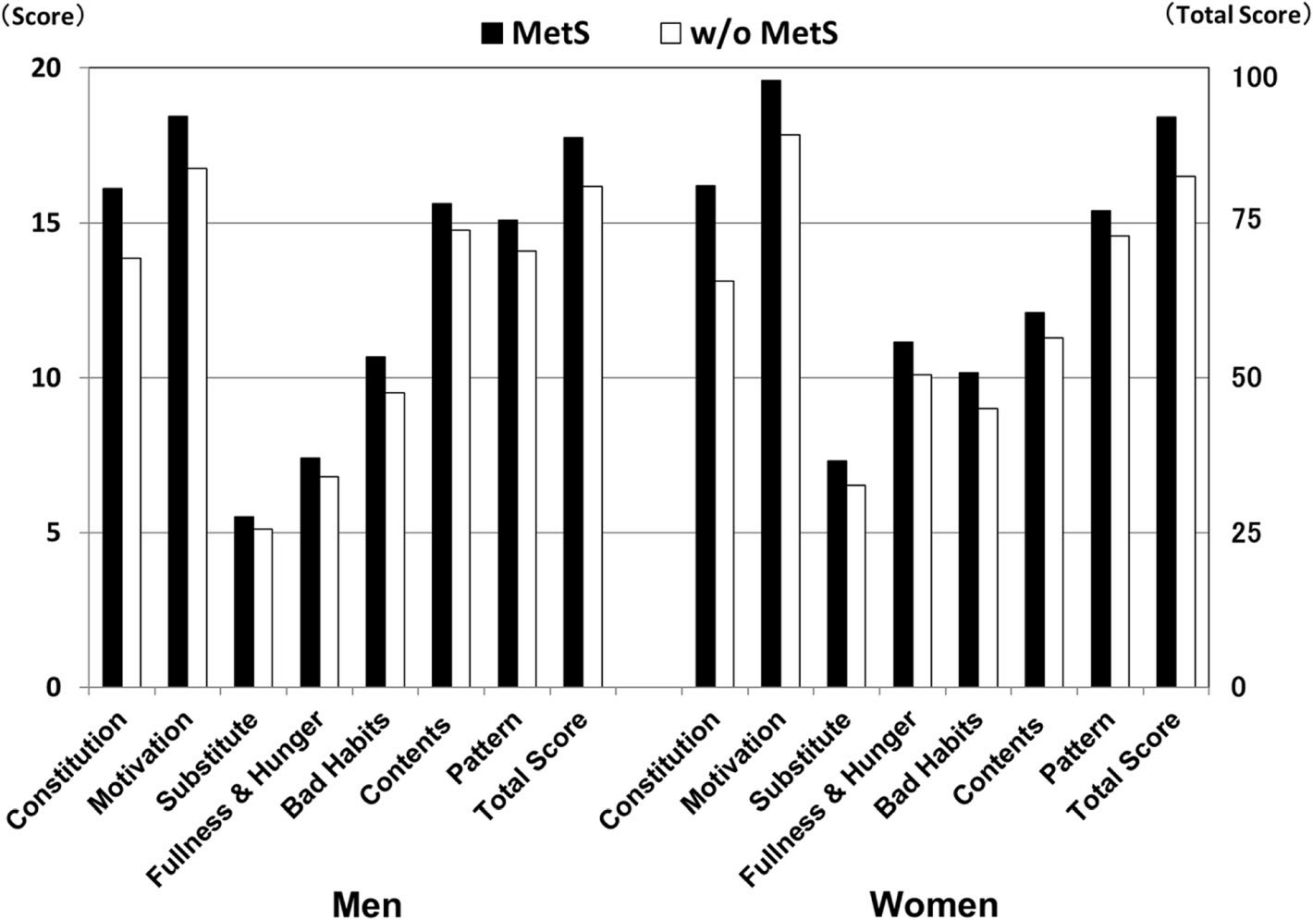
Associations between eating behavior and MetS. Eating behaviors were assessed by a questionnaire included 55 items by the Japan Society for the Study of Obesity. Scores were calculated based on a 4-point Likert scale for the following eight eating behavior categories:. 1 Constitution: perception gap about constitution and weight. 2 Motivation: motivation for eating. 3 Substitute: substitute eating (e.g., emotional eating). 4 Fullness and hunger: perception gap about feeling of fullness and hunger. 5 Bad habits: bad eating habits. 6 Contents: contents of diet. 7 Pattern: eating pattern. 8 Total score. The scores of all the categories in eating behavior were significantly worse in subjects with MetS than those without MetS (*p* < 0.05). MetS: metabolic syndrome, w/o: without

Besides, we analyzed the associations between eating behavior and MetS according to eight categories of the questionnaire. The scores of all the eating behavior categories were significantly worse in subjects with MetS than those without MetS (Fig. 2).

In correlation analysis among basic characteristics, dietary intake, and eating behavior, nearly all the correlation coefficients were less than 0.3. Exceptionally, positive correlations were found between age and energy-adjusted calcium (in both sexes), protein, sodium, potassium, and vitamin B_2_ in women. In contrast, negative correlations were found between the eating behavior scale of contents of meals and energy-adjusted potassium and calcium in women (data not shown).

## Discussions

The present study’s main results were (1) dietary intakes in the subjects with Mets were not higher than in those without Mets, except some nutrients and foods, and (2) eating behaviors were significantly worse in subjects with MetS than without MetS.

MetS is one of the most important problems of Japanese health policy. The prevalences of strongly suspected MetS were 24.7% in men and 9.4% in women in the 2012 National Health and Nutrition Survey (NHNS), Japan [21]. A higher prevalence of metabolic syndrome was reported recently (30.4% in men and 11.9% in women, 2018 NHNS). Our study presented a lower prevalence of MetS in our subjects. The difference in prevalence between our study and NHNS was attributable that the prevalence from NHNS was estimated from limited biomarkers and medical history. In the diseases, according to MetS, the most dominant disease was hypertension in our subjects. However, the prevalence of hypertension or high blood pressure (> 140 mmHg) in our subjects was lower than that in NHNS, despite higher sodium intake in our subjects than that in NHNS.

Previous epidemiologic cohort studies have been implemented to explain the correlation between food intake patterns and metabolic risk factors such as hypertension, obesity, and blood lipid profiles [22]. The scientific evidence has consistently shown [3, 4] as follows: (1) weight loss through an energy-restricted diet together with increased energy expenditure through physical activity contribute to the prevention and treatment of MetS [23]. (2) A Mediterranean-type diet, which refers to the traditional dietary pattern of countries in the Mediterranean basin, with or without energy restriction, is an effective treatment component [24]. (3) Other dietary patterns (Dietary Approaches to Stop Hypertension (DASH) [25], new Nordic [26], and vegetarian diets [27]) have also been proposed as alternatives for preventing MetS. However, Japanese diet has been not established yet.

Partly as a result of the Japanese population’s long life expectancy, dietary habits in Japan have been of interest to researchers from other countries [28]. Recent Japanese diets typically include high intakes of refined grains (mainly white rice), seaweeds, vegetables, fish, soybean products, and green tea, as well as low intakes of whole grains, processed meat, nuts, and soft drinks [20]. An answer was suggested in a review article. They said that the typical Japanese diet as characterized by plant food and fish, and the modest Westernized diet such as meat, milk, and dairy products might be associated with longevity in Japan [29]. Our subjects in the present study had a higher intake of energy, major nutrients, and foods for almost all food groups than the intake reported in 2012 NHNS, Japan, except for meats in both sexes and beverages in women. Dietary patterns of our participants may well adapt to Japanese healthy diet.

Sakata’s eating behavior scale was developed to use for the treatment of obesity in a clinical site. Our result that the subjects with MetS showed worse scores was assumed. The difference between subjects with MetS and without MetS withdrew after adjusting by BMI or weight. In our previous intervention study, we found that the behavior change was more important than food intake [13]. In this cohort, we will assess the subjects’ dietary modification during the follow-up period using this scale.

Some limitations of the present study need to be addressed. First, this study was a cross-sectional analysis. Thus, we were only able to suggest, and not determine, the causal direction in the association of eating behavior and dietary intake with MetS. The subjects with MetS may consciously decrease their dietary intakes through recognition of their obesity or hypertension previously. However, the eating behavior score in the subjects with MetS was obviously worse, so we did not expect their self-improvement of lifestyle, including diet. Second, physical activity is an important factor for weight loss and prevention of MetS as well as diet [30, 31], but we did not consider that factor in the present study. We assessed the participants’ physical activity levels by triaxial accelerometry (Actimarker EW4800, Panasonic Electric Works, Newark, NJ) and some self-administered questionnaires. Unfortunately, we have not finished calculating all parameters for physical activity yet. Next, in analyzing the study, we can use the data of the physical activity. Although smoking and alcohol drinking have been reported to be related to MetS, especially in men [32], smoking and alcohol drinking data were not available, and the analysis could not be adjusted for smoking and alcohol drinking as potential confounding factors. In near future, smoking and alcohol drinking data of this cohort will be available. Finally, the present study’s generality is limited because the participants of this study were healthy community residents seen for general health checkups every year. Assuming many people with metabolic syndrome were excluded because of their comorbidities, the prevalence of metabolic syndrome, particularly in women, was very low.

Many epidemiologic cohort studies have been implemented to develop adequate dietary behavior for MetS prevention. Nevertheless, there are inconsistencies and gaps in the evidence, and additional research is needed to define the most appropriate therapies for MetS.

We have been going on analyzing data of this cohort retrospectively and prospectively.

## Conclusions

In this baseline study, dietary intake differences between subjects with Mets and without Mets were small. The scores of all the eating behavior categories were obviously worse in subjects with MetS than without MetS. It was suggested that the problem lay in the quality of diet, not in the quantity, caused by bad eating habits. The potential influence of eating behavior and nutritional intake on MetS was presented in men and women.

## Supplementary Information

**Additional file 1:.** Sakata’s eating behavior questionnaire.

## Data Availability

Data sharing is not applicable to this article as no datasets were generated during the current study.

## Abbreviations

MetS: Metabolic syndrome
BMI: Body mass index
SCOP: Saku Control Obesity Program
OGTT: Oral glucose tolerance test
VFA: Visceral fat area
CT: Computed tomography
HbA1c: Hemoglobin A1c
HDL: High-density lipoprotein
LDL: Low-density lipoprotein
JDS: Japan Diabetes Society
NGSP: National Glycohemoglobin Standardization Program
HOMA-IR: Homeostasis model assessment of insulin resistance
BDHQ: Brief-type self-administered diet history questionnaire
DHQ: Self-administered diet history questionnaire
ANCOVA: Analysis of covariance
NHNS: National Health and Nutrition Survey
DASH: Dietary Approaches to Stop Hypertension
