# Supplementary material for: The associations of eating behavior and dietary intake with metabolic syndrome in Japanese: Saku cohort baseline study
Source: J Physiol Anthropol. 2020 Dec 14;39:40. doi: 10.1186/s40101-020-00250-w (PMC7734750; doi:10.1186/s40101-020-00250-w)
Supplement: Supplementary file 1 — Additional file 1:. Sakata’s eating behavior questionnaire. [file 40101_2020_250_MOESM1_ESM.docx]

Appendix 1. Sakata’s eating behavior questionnaire

| Question | | Question number from Manual of Obesity |
| --- | --- | --- |
| 1 | I often have a midnight snack. | 4 |
| 2 | I am a night person. | 18 |
| 3 | I don’t have a meal regularly. | 27 |
| 4 | I often eat between meals. | 21 |
| 5 | I don’t have enough time to eat. | 47 |
| 6 | I have dinner late. | 37 |
| 7 | I don't eat breakfast. | 48 |
| 8 | I’m often told I eat a lot. | 8 |
| 9 | I can eat my favorite foods right after eating. | 13 |
| 10 | I don’t feel satisfied unless I eat full. | 15 |
| 11 | I regret after I eat a lot. | 32 |
| 12 | I can’t sleep when I feel hungry. | 39 |
| 13 | I think about next meal right after eating. | 45 |
| 14 | I often eat snacks. | 11 |
| 15 | I like strong seasoning. | 14 |
| 16 | I often eat fast food like hamburgers. | 30 |
| 17 | I like greasy food. | 43 |
| 18 | I like noodles. | 19 |
| 19 | I often eat sweet pastries. | 40 |
| 20 | I love sweets. | 52 |
| 21 | I tend to eat left-over food because I don’t want to waste. | 12 |
| 22 | I tend to eat when I am irritated or stressed. | 16 |
| 23 | I always keep food around. | 23 |
| 24 | I tend to eat when I see others eating. | 24 |
| 25 | I tend to eat fruits and sweets when I see them. | 34 |
| 26 | I always gain weight whenever I take long holidays. | 20 |
| 27 | I tend to eat something when I have nothing to do. | 31 |
| 28 | I believe myself to gain weight more easily than others. | 42 |
| 29 | I believe myself to gain weight even by drinking water. | 22 |
| 30 | I eat fast. | 1 |
| 31 | I eat as putting food into my mouth one after another. | 55 |
| 32 | I don’t chew well. | 25 |
| 33 | I stuff food into my mouth. | 41 |
| 34 | I tend to order more than I can eat. | 28 |
| 35 | I cannot help buying more food than I need. | 33 |
| 36 | I cannot help cooking more than enough. | 38 |
| 37 | I believe that I gain weight because I like sweets. | 2 |
| 38 | I often buy at convenience stores. | 3 |
| 39 | I eat a lot of good food for dinner compared with other meals. | 35 |
| 40 | I gain weight because I don’t have sufficient physical activities. | 36 |
| 41 | I feel uncomfortable unless I keep enough food in a refrigerator. | 5 |
| 42 | When I find something good at grocery stores, I buy it unexpected. | 44 |
| 43 | I often drink beer. | 46 |
| 44 | I am not satisfied when a very few-food items are served at dinner. | 17 |
| 45 | I don’t have a sense of hunger and fullness. | 49 |
| 46 | I have many social occasions to eat. | 50 |
| 47 | I don’t lose weight although I don’t eat much. | 51 |
| 48 | I tend not to be hungry before meals. | 53 |
| 49 | I eat meat a lot. | 54 |
| 50 | I believe that I gain weight because I lie down soon after eating. | 6 |
| 51 | I have many occasions to attend drinking parties. | 7 |
| 52 | I get irritated when I’m hungry. | 9 |
| 53 | I eat well even if I have a cold. | 10 |
| 54 | I eat more Western food than Japanese food. | 29 |
| 55 | I often eat out and have food delivered. | 26 |

**Calculation of each category score by sex**

| Category | "Question number from Manual of Obesity" included in each category | Maximum score of each category |
| --- | --- | --- |
| Men |  |  |
| 1 Perception gap about constitution and weight | 2, 6, 10, 22, 36, 42, 51 | 28 |
| 2 Motivation for eating | 12, 13, 24, 28, 33, 34, 38, 44, 45, 50 | 40 |
| 3 Substitute eating (e.g. emotional eating) | 5, 16, 23, 31 | 16 |
| 4 Perception gap about feeling of fullness and hunger | 9, 15, 32, 53 | 16 |
| 5 Bad eating habits | 1, 8, 25, 41, 55 | 20 |
| 6 Contents of diet | 11, 14, 26, 29, 30, 40, 43, 52, 54 | 36 |
| 7 Eating pattern | 4, 7, 20, 21, 27, 35, 37, 47 | 32 |
| 8 Total score | Sum of above all items | 188 |
|  |  |  |
| Women |  |  |
| 1 Perception gap about constitution and weight | 2, 6, 10, 22, 36, 42 | 24 |
| 2 Motivation for eating | 12, 13, 17, 24, 28, 33, 38, 44, 50 | 36 |
| 3 Substitute eating (e.g. emotional eating) | 5, 16, 23, 31 | 16 |
| 4 Perception gap about feeling of fullness and hunger | 9, 15, 32, 39, 49, 53 | 24 |
| 5 Bad eating habits | 1, 8, 25, 41, 55 | 20 |
| 6 Contents of diet | 3, 19, 26, 30, 40, 43, 54 | 28 |
| 7 Eating pattern | 4, 18, 20, 21, 27, 35, 37, 48 | 32 |
| 8 Total score | Sum of above all items | 180 |
